# Translation, linguistic validation and reliability of FACT-H&N questionnaire in Oral Cancer patients in Sudan

**DOI:** 10.1186/s41687-022-00507-1

**Published:** 2022-09-16

**Authors:** M. El Sheikh, A. Suleiman, A. Satti, E. M. O’Sullivan

**Affiliations:** 1 Oral & Maxillofacial Surgery Department, Faculty of Dentistry, University of Khartoum, Khartoum, Sudan; 2 Computing and Research Department, Khartoum Teaching Dental Hospital, Ministry of Health, Khartoum, Sudan; 3 Oral & Maxillofacial Surgery Department, Cork University Dental School & Hospital, UCC, Cork, Ireland

**Keywords:** Arabic, FACT-H&N, Quality of life (QoL), Sudan, Oral cancer, Head and neck cancer, Translation

## Abstract

**Background:**

This study aimed to translate and validate an Arabic version of the Functional Assessment of Cancer Therapy Head and Neck Scale (FACT-H&N, v-4) for use among Sudanese oral cancer patients.

**Methods:**

The instrument underwent translation and validation following the standard FACT translation methodology. The translated instrument was pre-tested for face validity and content validity using semi-structured, in-depth interviews with ten oral cancer patients to assess acceptability. The questionnaire was pilot tested with 60 patients; reliability was tested for internal consistency with Cronbach’s alpha while construct validity was tested using ‘known-group validity’.

**Results:**

The pre-test study revealed no major issues, apart from a reluctance to answer questions on sexual satisfaction. The FACT-H&N demonstrated good internal consistency, it considered five particular constructs: PWB, SWB, EWB, FWB and FACT-H&N, their Cronbach’s α values were positive and close to 1 with values of 0.85, 0.788, 0.86, 0.895 and 0.703 respectively, indicating that the questionnaire was valid and the responses consistent. Sixty patients were asked the global health-related quality of life item, 36.3% rated their QOL as very good or good (36.3%), while 41.7% rated it as average, and 21.7% as poor or very poor. Then FACT subscale mean scores were tabulated against three categories; patients with very poor/poor recorded significantly lower scores indicating construct validity. Some psychometric properties were consistent with other FACT-H&N translations such as the Chinese, French, Pakistani and Malaysian.

**Conclusions:**

This study validates the Arabic version of the FACT-H&N. It is a reliable tool and, will assist further QoL research in other Arabic-speaking countries.

## Plain English Summary

While survival rates are routinely used to evaluate treatment outcomes in oral cancer, increased understanding of the human cost of this disease highlights the need to also consider the impact of the disease and therapy on patients’ quality of life (QoL). Measurement of quality of life outcomes requires a validated QoL questionnaire with proven applicability in that specific context. Little research has been undertaken on the QoL of cancer patients in Sudan and no published research exists to date on the QoL of Sudanese oral cancer patients. While an Arabic version of the FACT-G general questionnaire exists, the FACT-H&N module was not previously available in Arabic. This paper describes the translation of the FACT-H&N from English into Arabic to suit Sudanese patients and other Arabic-speaking patients, with a detailed pre-test review by 10 patients, a pilot study with 60 patients and further validation with 130 patients. This study represents the first QoL research undertaken in oral cancer patients in Sudan and provides some insight into the impact of oral cancer on their lives. Findings from this study indicate that the Arabic version of the FACT-H&N questionnaire is a valid, reliable and acceptable method for assessing health related QoL in Arabic-speaking Sudanese oral cancer patients. This work will benefit future researchers working with Arabic-speaking oral cancer patients.

## Background

Collection of site-specific, multi-dimensional patient-reported (QoL) outcomes is now established as a key component in overall cancer care [1]. This is particularly relevant for patients with oral cancer, as this disease may impact negatively on many quintessential aspects of life—speaking, eating, swallowing, and aesthetics. Surgery may require the loss of important functional units e.g. tongue/palate, while multimodality care has greatly exacerbated therapeutic morbidity. Furthermore, oral cancer patients frequently experience a wide range of life-altering, life-long disease and treatment-related side-effects and distressing complications which may have profound physical, emotional and social effects [1–6]. Therefore, treatment decisions require careful consideration of the potential risks and benefits of each therapeutic option, bearing in mind the QoL impact of the disease and of any proposed treatment plan.

Sudan is a multi-cultural nation with a population of over 45 million. Most of the population are Muslim and 55% live at or below the poverty level. The official language is Arabic and adult literacy levels are relatively low at c.60% [7, 8]. While oral cancer is one of the ten most common cancers in Sudan [9, 10], an extensive literature review failed to reveal any published quality of life studies for oral cancer patients in that region. A prospective study on the QoL of oral cancer patients in Sudan was therefore undertaken between 2013 and 2016 using the Functional Assessment of Cancer Therapy (FACT) questionnaire developed by Cella et al. [11]. While the general FACT questionnaire (FACT-G), had been previously translated into Arabic, the Head and Neck specific subscale (FACT-H&N) was not available in this language. This paper describes the methodology used to translate and cross-culturally validate an Arabic version of the FACT-H&N for use in patients with oral cancer in Sudan and other Arabic-speaking populations.

## Methods

This study was undertaken as part of a longitudinal, prospective study on survival rates and QoL for oral cancer patients at Khartoum Teaching Dental Hospital (KTDH), Sudan from January 2013 to December 2016. KTDH is the main Maxillofacial surgical centre, to which the majority of oral cancer cases from all over Sudan are referred. Ethical approval was obtained from the Ministry of Health Ethics Committee, Sudan. While several QoL instruments exist, the FACT (V-4) QOL instrument was chosen as it has been widely validated and translated into many languages including Arabic, and its reliability had been previously confirmed by many researchers [12–15]. Furthermore, the questionnaire is short, easily completed and feasible for busy clinical settings. The modular construction of FACT allows comparison across cancer diagnoses while still probing issues specific to oral cancer. It consists of four subscales, including physical, social, functional, and emotional well-being issues. Response options comprised a 5-point Likert scale (‘not at all’, ‘a little bit’, ‘somewhat’, ‘quite a bit’, and ‘very much’, scored from 0 to 4), which indicates ‘how true’ the patient's response over the past seven days, with higher FACT subscale and summary scores denoting better QoL. The additional 12-item Head & Neck (FACT-H&N) specific subscale was not available in Arabic at that time.

*Participants* Formal sample size calculation was not undertaken, the sample size was determined by the number of patients presenting during the recruitment period. All new patients with histologically proven oral cancer (ICD-10, C00, C02-08, C14.8, C31.1, C41.1), attending KDTH between January 2013-December 2016, who satisfied the inclusion criteria were consented and enrolled in the prospective study. Basic demographic and clinical details were collected as part of this study, including age, gender, marital status, living arrangements, habits, tribe, tumour stage, type and chronic illness profile. Of these, 130 agreed to participate in the QoL study, with 60 participating in the initial pilot study. A single interviewer identified and approached all newly diagnosed patients explained the purpose of the study, and following consent administered and collected all questionnaires. As literacy levels in Sudan are relatively low, the Questerview technique was used where required [16].

*Inclusion criteria* Adult patients (18 years and above) with histologically confirmed oral cancer, who were able to speak/understand Arabic and who had not yet received any oral cancer therapy. Patients deemed unable to understand or respond to the QoL questionnaire or unable to speak/understand Arabic were excluded.

*Translation process* Translation to Arabic and validation steps were undertaken with the advice and consent of the original developers to adapt it for Sudanese oral cancer patients, following standard FACT translation methodology [15]. Steps involved: 1) Forward translations from English to Arabic by two expert translators from Khartoum University, working independently, 2) Reconciliation of these two forward translations by a third translator, 3) Back translation into English by a fourth translator, 4) Review and finalization by a fifth translator, 5) Proof-reading the Arabic FACT-H&N questionnaire by the bi-lingual, bi-cultural researcher, 6) lastly, the Arabic translated FACT-H&N was pre-tested for face validity and content validity, then cross-culturally adapted for the Sudanese context in terms of its conceptual and operational equivalence. Pre-testing was undertaken by the main researcher using semi-structured, face-to-face in-depth interviews with a purposive sample of ten oral cancer patients of different ages, cancer stages and cancer types. These ten patients completed the translated questionnaire, and then answered the Patient Interview Form (PIF), a cognitive debriefing script provided by the original developers, to assess acceptability and to see whether any items were confusing or upsetting. The item on sex satisfaction was considered unacceptable by most patients and had a higher rate of missing data. All other items were deemed clear, acceptable and understandable.

### Pilot study

Sixty newly diagnosed oral cancer patients were enrolled in the pilot study; they completed the baseline assessment, and a test-retest seven days later, to assess the feasibility, reliability, validity and internal consistency, i.e., psychometric properties, of the FACT-H&N scale and the patients’ response to the translated Arabic version. FACT summary scores included the general module (FACT-G), head and neck specific module (FACT-H&N), FACT symptom-index score (FHNSI) which is a subset of the specific FACT-H&N module, and the FACT-H&N-Trial Outcome Index (FACT-H&N TOI) which is a combination of the functional, physical and head and neck subscales, and the head and neck symptom index (FHNSI).

Instrument reliability was evaluated by the Test-Retest Reliability at baseline and after seven days using the Intra-Class Correlation Coefficient (ICC), while the internal consistency reliability was evaluated using Cronbach’s α coefficient for the pilot group. Construct validity was assessed using the known group’s validity by the analysis of variance (ANOVA) test: pilot study patients were asked the global health-related QoL item: “how do you rate your health-related quality of life in the past 7 days”? The FACT summary and subscale mean scores were tabulated against the three categories of the global HRQOL item (very poor/poor, average and good/very good).

Furthermore, the patients who took part in the pilot study were included in a longitudinal prospective study. A total of 130 patients were enrolled, and they were assessed for their HRQOL at four data collection points: at diagnosis (baseline), and one, three and 12 months later after commencing treatment. Once again, the cross-sectional construct validity was assessed using known group validity. All 130 patients were categorized at diagnosis (baseline) into early (I/II) or late/advanced disease (stage III/IV). FACT summary and subscale mean scores for early and late stages were compared using the independent t-test. Convergent validity was assessed using the correlation matrices for FACT summary scale and sub-scales using Pearson’s correlation co-efficient.

Missing responses were managed by pro-rating these scores, as recommended in the FACT manual. The sum of each FACT-G module (27 items) was calculated. Derivatives of FACT scores were calculated. All statistical tests were done using SPSS® version 22.0; *p* < 0.05 was considered statistically significant.

## Results

### Demographics

A sample of 130 patients completed this study. The overall male to female ratio was 2.1–1; the mean age was 56.5 SD ± 14.2 years. Most patients were married (80%) and all lived with their families or extended families. The vast majority (75.4%) presented with advanced disease (stage III/IV), while only 3.1% presented with stage I (T1N0). SCC was the dominant histology (77.7%), followed by salivary malignancies (15.3%), and rarer malignancies (7%). The most common tumour sites were: gum (C03.1, 32.3%), tongue (C02, 18.5%), palate (C05, 13.1%) and malignant neoplasms not otherwise specified NOS (C06.0–9, 18.5%).

### Reliability

Test-Retest Reliability was assessed using ICC (Table 1). ICC value of the total outcome index (FACT H&N TOI) was the highest at 0.924, while the other two sub-scales with an ICC > 0.9, included FACT Total (0.903) and functional well-being (FWB), (0.914). The lowest ICC value was for emotional well-being (EWB), 0.793.

**Table 1:** Test-retest reliability results using ICC—Higher scales indicate better QOL

Sub-Scale (No. of items)	Descriptive statistics						
	Possible score		Observed Score		Baseline sample Mean	SD	ICC
	Min	Max	Min	Max			
PWB (7)	0	28	0	24	13.1	6.1	.818
SWB (7)	0	28	8	28	21.0	5.3	.849
EWB (6)	0	24	2	24	16.4	6.0	.793
FWB (7)	0	28	2	28	15.1	6.7	.914
FACT-H&N (12)	0	40	24	100.8	65.6	20.6	.883
FACT G (27)	0	108	4.2	31.8	18.2	5.5	.887
FACT H&N TOI (26)	0	96	9.2	77.2	46.3	16.5	.924
FACT Total (39)	0	148	31.8	127.5	83.8	24.7	.903

Validity and internal consistency of the Arabic FACT-H&N questionnaire were evaluated using Cronbach’s α coefficient for the 60 pilot study patients (Table 2). The questionnaire considered five particular constructs: PWB, SWB, EWB, FWB and FACT-H&N. Cronbach’s α values for all constructs were positive and close to 1, indicating that the questionnaire was valid and the responses consistent. The internal consistency of FWB was higher than the other four constructs, with a value of 0.895.

**Table 2:** Internal consistency results using cronbach’s alpha coefficient

Subscale	No. of items	Cronbach’s α
Physical (PWB)	7	0.850
Social (SWB)	7	0.788
Social (SWB) Q7 excluded	6	0.789
Emotional (EWB)	6	0.869
Functional (FWB)	7	0.895
Head & Neck subscale (FACT-H&N)	12	0.703

### Cross-sectional construct validity

The 60 pilot study patients were asked the HRQoL item:” how do you rate your health-related quality of life in the past 7 days”? Just over one-third rated their QoL as very good (2, 3%) or good (20, 33.3%), while 25 patients (41.7%) rated it as average, and one-fifth as poor (10, 16.7%) or (3, 5%) very poor. The FACT summary and subscale mean scores were then tabulated against these three categories. Patients with very poor/poor health related QoL recorded significantly lower scores for each FACT summary scale and the H&N subscale, indicating construct validity (Table 3). Similarly, baseline FACT summary and subscale mean scores by tumour stage for the full study group (n = 130), showed significantly lower FACT scores for those with advanced disease across all four FACT summary scales and for the physical, social and functional subscales, indicating a high degree of validity. The emotional subscale (EWB) showed a similar trend but was not statistically significant, (Table 4).

**Table 3:** Cross sectional validity results,

	Very poor/Poor	Average	Very good/Good	*P* value
No of patients (%)	13 (21.7)	25 (41.7)	22 (36.6)	

Summary mean scores (SD)	Mean (SD)	Mean (SD)	Mean (SD)	
PWB	7.9 (4.9)	15.0 (5.7)	16.7 (4.7)	0.001**
SWB	19.3 (6.0)	22.4 (3.6)	24.3 (3.9)	0.007**
EWB	12.9 (6.2)	18.0 (4.4)	21.0 (3.6)	0.001**
FWB	8.8 (4.5)	16.0 (6.3)	20.6 (5.0)	0.001**
FHNSI	13.2 (4.6)	19.4 (4.1)	21.0 (5.8)	0.001**
FACT G	49.0 (16.5)	71.4 (15.1)	82.5 (13.4)	0.001**
FACT H&N TOI	30.0 (12.1)	50.5 (13.4)	58.3 (12.9)	0.001**
FACT Total	62.2 (19.9)	90.8 (16.9)	103.5 (17.1)	0.001**

n = 60; **P < 0.05 One-way ANOVA

**Table 4:** Cross sectional validity results

Scale	Stage I&II	Stage III&IV
Number of patients (%)	32 (24.6%)	98 (75.4%)

Summary mean scores	Mean (SD)	Mean (SD)
PWB	18.84 (4.13)	13.40 (5.74)*
SWB	23.78 (4.31)	20.68 (5.42)*
EWB	16.72 (5.27)	16.36 (6.41)
FWB	19.53 (6.00)	14.68 (6.80)*
FHNSI	22.81 (4.94)	18.10 (5.81)*
FACT G	78.9 (14.7)	65.1 (20.5)*
FACT H&N TOI	61.19 (12.95)	46.18 (16.04)*
FACT Total	101.68 (18.05)	83.22 (24.49)*

n = 130; *p value < 0.05 independent t-test

### Convergent and discriminatory validity

Correlation matrices for the FACT summary scale and sub-scales using Pearson’s correlation coefficient were performed on baseline data from all 130 patients. All FACT summary scales correlated strongly with each other (r > 0.75), except for a moderate correlation (0.5 < r < 0.75) between FHNSI and FACT-G. The strongest correlation was seen in FACT-G and FACT Total (0.982). Summary scales FACT-H&N and FACT-H&N (TOI) showed convergent validity (r > 0.90), and little evidence of discriminant validity. Low correlations were noted between both the social and emotional subscales and the FHNSI (0.392 and 0.421 respectively) and also with the physical subscale (0.448 and 0.482 respectively), suggesting very low convergent but high discriminant validity between these subscales. High convergent validity was also seen between the functional and FHNSI, FACT-H&N (TOI), and FACT Total scales, (Table 5).

**Table 5:** Correlation matrices using Pearson’s correlation coefficient

	PWB	SWB	EWB	FWB	FHNSI	FACTG	FACT HN TOI	FACT total
PWB								
SWB	0.448**							
EWB	0.482**	0.518**						
FWB	0.733**	0.596**	0.656**					
FHNSI	0.688**	0.392**	0.421**	0.613**				
FACT G	0.810**	0.759**	0.809**	0.916**	0.644**			
FACT H&N TOI	0.905**	0.547**	0.595**	0.895**	0.857**	0.899**		
FACT Total	0.836**	0.722**	0.770**	0.906**	0.776**	0.982**	0.951**	

^**^ Correlation significant at 0.01 level, * Correlation significant at 0.05 level

## Discussion

While mortality and disease-free survival rates are routinely used to evaluate therapeutic outcomes in head and neck cancer (HNC), recognition of the bio-psycho-social impact of these tumours has highlighted the importance of including quality of life outcomes. However, QoL data are of little value unless they have been obtained using a validated questionnaire of proven applicability in the context concerned [12]. This paper describes the method used to translate and pilot test the FACT-H&N subscale from its original English language into Arabic using well-accepted, validated methods of translation, item review, expert input and deliberation. It assesses the reliability and validity of the Arabic FACT-H&N questionnaire which was cross-culturally adapted to meet the needs of the Sudanese population. This study also represents the first QoL study undertaken in HNC patients in Sudan.

The Arabic FACT-H&N demonstrated good internal consistency in the Sudanese pilot study using Cronbach’s α and proved to be valid with highly consistent responses. The Arabic FACT-H&N compares favourably with similar studies from a range of countries regarding internal consistency, (Table 6). The internal consistency of the Functional Well Being (FWB) construct was found to be higher than the other five subscales, similar to results from Pakistan, Malaysia and China. Moreover, the head and neck subscale showed good consistency, which is consistent with the Chinese version and higher than French, Pakistani and Malaysian versions.

**Table 6:** Comparisons between Sudanese Arabic FACT-H&N and other translations

Country and year	Sudan 2017	Pakistan 2014 [17]	China 2008 [18]	Malaysia 2011 [13]	India 2004 [19]	French 2004 [12]
Authors		Bilal et al.	Chang et al.	Doss et al.	Thomas et al.	Conroy et al.
Reliability Cronbach’s α						
Physical well being	0.850	0.92	0.79	0.82	0.75	0.87
Social well being	0.788	0.90	0.81	0.65	0.65	0.66
Emotional well being	0.869	0.72	0.72	0.71	0.71	0.68
Functional well being	0.895	0.96	0.88	0.87	0.87	0.84
Head & neck subscale	0.703	0.42	0.75	0.49	–	0.52

The self-rated global health related quality of life item “how do you rate your health related quality of life in the past 7 days” was an effective and true reflection/representation of the patient’s overall QOL. Similarly, close associations between global measures of health status and multidimensional scales were noted in HNC patients in Malaysia. This enabled the global item to be used to assess the multidimensional scales validity [13].

Analysis showed that the FACT-H&N summary scales and subscales were able to differentiate between early (I/II) and late (III/IV) tumour stages with different QoL scores, demonstrating good known group validity. The mean scores across all domains, apart from emotional well-being, were significantly higher in early stages compared to advanced stages, denoting better HRQoL in early disease. These findings were consistent with the psychometric properties of the cross-culturally adapted FACT-H&N. Similarly, reports from Spanish, Malaysian and Pakistani HNC patients indicate significantly lower QoL scores in late stage disease [13, 17, 20].

One of the essential domains impacting upon cancer patients’ QOL is emotional well-being [21]. Oral cancer patients face many challenges over the disease trajectory. The often onerous uphill journey may affect many aspects of their lives, leading them to face psychological distress, depression or anxiety [22, 23]. Surprisingly, in this study, mean scores in the emotional well-being domain were similar in patients with early (mean ± SD = 16.72 ± 5.27) and late stage disease (mean ± SD = 16.36 ± 6.41). This unexpected finding may be linked to the fact that QoL is affected by the intrinsic characteristics of each individual patient, namely their beliefs, expectations and experiences [5, 24]. The negligible difference in emotional well-being scores between early and late stage cases may be attributed to the fact that Sudanese patients, as Muslims, are strong believers in fate and destiny and their beliefs affect how they cope with disease. Strong family support is also a feature of patient care in Sudan. Similarly, oral cancer seemed to be borne silently by Malaysian patients who coped by opting not to focus on their illness [13].

## Conclusion

Measurement of health related QoL (HRQoL) is important to assess treatment outcomes, educate patients and clinicians about the full impact of treatment and to facilitate decision-making. This is particularly important for patients with oral cancer, considering the important functional and social role of the oral cavity and related structures. This research represents the first QOL study undertaken to date in oral cancer patients in Sudan and it provides an insight into the impact of oral cancer on the lives of these patients. This study shows that the Arabic version of the FACT-H&N questionnaire is a valid and reliable method for assessing HRQoL in Arabic-speaking Sudanese oral cancer patients which should benefit future researchers working with Arabic speaking HNC patients.

## Limitations

While the study adds to previous literature, some limitations must be acknowledged. Participation in this study was limited to a convenient sample of patients in the early treatment phase and did not include long-term survivors. While the sample size was adequate, further research with a larger sample size, including long-term HNC survivors is recommended.

## Data Availability

Available upon request at any time.

## Abbreviations

ANOVA: Analysis of variance test
EWB: Emotional Wellbeing
FWB: Functional Wellbeing
FACT-H&N: The functional assessment of cancer therapy head and neck scale
FACT-G: Functional assessment of cancer therapy general module
FACT-H&N TOI: FACT-H&N-trial outcome index
FHNSI: FACT symptom-index score
HRQoL: Health-related quality of life
HNC: Head and neck cancer
ICC: Intra-class correlation coefficient
KTDH: Khartoum Teaching Dental Hospital
PIF: Patient interview form
PWB: Physical wellbeing
QoL: Quality of life
SWB: Social wellbeing
